# Lessons from Community Mental Health to Drive Implementation in Health Care Systems for People with Long-Term Conditions

**DOI:** 10.3390/ijerph110504714

**Published:** 2014-04-30

**Authors:** Michele Tansella, Graham Thornicroft, Heidi Lempp

**Affiliations:** 1 Department of Public Health and Community Medicine and WHO Collaborating Centre for Research and Training inn Mental Health and Service Evaluation, University of Verona, Verona 37134, Italy; 2 Health Service and Population Research Department, Centre for Global Mental Health, Institute of Psychiatry, King’s College London, De Crespigny Park, London SE5 8AF, UK; E-Mail: graham.thornicroft@kcl.ac.uk; 3 Clinical Trails Group/Academic Rheumatology, School of Medicine, King’s College London, Weston Education Centre, 10, Cutcombe Rd., London SE5 9RJ, UK; E-Mail: heidi.lempp@kcl.ac.uk

**Keywords:** long term conditions, chronic disorders, mental health care and services, community mental health, integrated care

## Abstract

This paper aims to identify which lessons learned from the evidence and the experiences accruing from the transformation in mental health services in recent decades may have relevance for the future development of healthcare for people with long-term physical conditions. First, nine principles are discussed which we first identified to guide mental health service organisation, and all of which can be potentially applied to long term care as well (autonomy, continuity, effectiveness, accessibility, comprehensiveness, equity, accountability, co-ordination, and efficiency). Second, we have outlined innovative operational aspects of service user participation, many of which were first initiated and consolidated in the mental health field, and some of which are now also being implemented in long term care (including case management, and crisis plans). We conclude that long term conditions, whether mental or physical, deserve a long-term commitment from the relevant health services, and indeed where continuity and co-ordination are properly funded implemented, this can ensure that the symptomatic course is more stable, quality of life is enhanced, and the clinical outcomes are more favourable. Innovations such as self-management for long-term conditions (intended to promote autonomy and empowerment) need to be subjected to the same level of rigorous scientific scrutiny as any other treatment or service interventions.

## 1. Introduction

In most high income countries there has been a profound transformation in mental health care over the last 30 years. A system that was based in large and often remote psychiatric hospitals has been reconfigured into a far more complex pattern. This new system includes different types of community mental health teams, a range of treatment, rehabilitation, employment and residential care facilities in the community, and liaison with primary care, alongside the continuing provision of a relatively small number of acute hospital beds. Further, there is an increasingly central role for service users in planning and/or in providing services, with close working with patient and carer advocacy groups [1].

In this paper we offer a personal reflection upon these historic changes, in particular those affecting people with long-term disorders. We aim to identify which lessons learned from the evidence and the experiences accruing from this transformation in mental health services may have relevance for the future development of healthcare for people with long-term physical conditions (which we prefer to the term ‘chronic disorders’ which tends accentuate therapeutic pessimism). We use a public health approach, by which we mean largely state funded healthcare systems, for both mental and physical healthcare services. We appreciate therefore that these lessons may not fully apply to systems which are largely or substantially privately funded or provided.

Long term conditions are increasingly common. In England, for example, more than 15 million people (30% of the population) have one or more long-term conditions, a figure expected to increase to 18 million by 2025 [2]. These figures include people with a range of conditions that can be managed, rather than cured, by medication and/or other treatments and therapies, such as diabetes, arthritis, asthma and hypertension. To this we can add conditions such as HIV/AIDs and certain cancers, which have not traditionally been considered long-term conditions, but which are increasingly regarded as such as therapeutic outcomes improve over time. 

Many mental health problems can also be considered long-term conditions, nevertheless for the sake of clarity we use the term ‘long-term conditions’ here to refer to physical health conditions alone. It is also important to recognise that long term mental and physical disorders often co-occur. In England, for example, 30% of people with a long-term condition also have a mental health problem, and 46% of people with a mental health problem have a long-term condition [2]. Nevertheless, comorbidity is not the main focus of this paper. Rather we shall discuss the usual configuration of healthcare in most countries worldwide, namely the separation between services for people with physical and mental conditions. 

A word of caution is needed. We are aware that transferring lessons learned in one historical and geographical context to another may be a complex process. Many factors influence which innovations or interventions may be congruent with the healthcare system, the socio-cultural and economic context, and determine whether a health service component may have a degree of fit to a new context, if it is locally adapted. But there will also be cases in which no such adaptation is practicable. We therefore offer these ‘lessons’ in the spirit of ideas which may be of use to the reader, depending upon local circumstances. 

## 2. Establishing Fundamental Principles, Followed by Planning and Implementing Service Changes for People with Long-Term Conditions

The context for the provision of treatment and care for people with long-term mental disorders in many high income countries has been that, until the last generation, many were kept as long-term in-patients over many decades. Throughout Western Europe, for example, most of these beds, and many of these hospitals, have been closed and now subsequent generations of people with severely disabling mental conditions are mostly treated outside hospital [3]. In designing community-based mental health services, we have proposed a series of 9 principles to guide planning decisions [4]. These principles were initially considered as orthogonal, or mutually independent, but experience has demonstrated to us that in fact some of these principles may be either positively or negatively correlated with each other. 

These nine fundamental principles can also be used to guide decisions related to long-term conditions. However a number of caveats need to be kept in mind. First, there has not been the need to de-institutionalise care for people with most long term conditions (with the exception of tuberculosis). Second, historical and current levels of funding mean that the level or coverage (meaning the proportion of people with a particular diagnosis who actually receive treatment) shows an ‘inverse care law’ in that in many high income countries about 75% of people with long term conditions are treated, whereas only 25% of people with mental disorders receive care [5,6]. Third, demand for mental health care may be substantially impeded by stigma-related factors, including lack of knowledge of locally available services, as well as expectations of low quality treatment, and of stigmatising reactions to a person who has received psychiatric treatment [7,8].

### 2.1. Autonomy

This is a patient-level characteristic consisting of the ability to make independent decisions and choices, alongside the presence of continuing symptoms or disabilities. This principle has been put into practice in mental health services, for example, using such innovations as decision support tools, crisis plans, advanced statements and forms of self-management [9,10]. This is highly relevant for long-term conditions such as rheumatoid arthritis where self-management is now well developed, for example in cognitive-behaviour therapy self-management groups for chronic fatigue or for flare-ups (sudden periods of clinical deterioration) of rheumatoid arthritis [11,12]. There is accumulating evidence that self-efficacy, and psychological well-being can be enhanced by self-management for people with arthritis [13]. A review of self-management approaches for 28 long term disorders shows that: such programmes can have a beneficial effect on the wellbeing of participants in the short term and achieve increased knowledge, self-efficacy and use of self-management behaviours, and this was particularly the case for people with diabetes mellitus and hypertension [14], although the quality of the relevant research studies is not consistently strong. 

### 2.2. Continuity

This refers to ability of health services to offer interventions which provide coherence between different members of staff, and across different clinical teams, in the short-term (*cross-sectional continuity*), and which offers a stability of relationships and therapeutic knowledge, in the context of a regular series of contacts, in the long term (*longitudinal continuity*). The importance of continuity of care for people with long term mental disorders has been understood for over a decade [15,16], with a range of methods implemented to enhance continuity by the use of case managers [17]. Case management is now also increasingly recognised in relation to long-term conditions [18]. To measure the impact of continuity of care, Cowie *et al.* have applied a multi-dimensional model to people with seven long-term conditions (arthritis, coronary heart disease, stroke, hyper-cholesterolaemia hypertension, diabetes mellitus or COPD) [19]. They found that patients’ experiences of health care can be understood in terms of both relational and management continuity’ [19].

### 2.3. Effectiveness

We have defined effectiveness as ‘the proven, intended benefits of services provided in real life situations’ [4]. This is now the central tenet of evidence-based medicine and a large series of systematic review referring to long term conditions are available at evidence repositories such as the Cochrane Library. A recent systematic review suggests that the range of treatments available for mental and physical conditions, including those which may have long-term consequences, have essentially similar effect sizes [20]. Nevertheless there is now a robust debate on *who* should decide which outcomes are most important, and who should design scales to measure these criteria, as we discuss below in the section on patient participation.

### 2.4. Accessibility

This is a service characteristic, experienced by service users and their carers, which enables them to receive appropriate care where and when it is needed. The actual location of care will vary according to where healthcare (at the appropriate level of expertise) is available, and according to the complexity of individual cases. For example, where primary care staff are sufficiently trained and have access to relevant investigations and equipment, then there may be little need to refer cases to secondary (specialist) care. At the same time a person with highly unstable diabetes mellitus may need to have less accessible but more specialised care. This is an example of a trade-off (or offset) between principles, which may in some cases be competitive rather than complementary [21].

### 2.5. Comprehensiveness

This is a service characteristic with two dimensions: by *horizontal comprehensiveness* we mean how far a service extends across the whole range of severity of mental illnesses; by *vertical comprehensiveness* we mean the availability of the basic components of care (out-patient and community care; day care; acute in-patient and longer-term residential care; interfaces with other services), and their use by prioritised groups of patients. For people with long term conditions, there is evidence of very considerable variations in people’s experiences and in the quality of the care they receive. For rheumatoid arthritis (RA), for example, many people report that: they wait too long for referral to a specialist, the care they receive over-emphasises pharmacological treatment at the expense of psycho-social interventions [22].

### 2.6. Equity

This refers to the fair distribution of resources and the basis upon which competing needs can be prioritised. For example, it is clear across the world that extremely low proportions of national healthcare budgets are spent on mental health services, regardless of the levels of need or the global burden of disease [23]. In relation to long-term care, a number of factors need to be kept in mind, including whether patients of different sub-groups of the population have reasonable and equal access to new forms of treatment or self-management [24], for example by ethnicity [25], or by age [26]. There may also elements of competition, not only *between* these principles, but also *within* aspects of the same principle. For example, issues of equity of choice of treatments may conflict with equity of outcomes across the whole patient population [27].

### 2.7. Accountability

This principle is a function which consists of complex, dynamic relationships between health services and patients, their families and the wider public, who all have legitimate expectations of how the service should act responsibly. In fact this principle applies in the same way to both mental [28,29] and physical healthcare [30] in that both need to be managed in a way that shows the relevant funders that resources are being spent responsibly, fairly and effectively.

### 2.8. Co-Ordination

A service characteristic which is manifested by coherent treatment plans for individual patients. Each plan should have clear goals and should include interventions which are needed and effective: no more and no less. By *cross-sectional* co-ordination we mean the co-ordination of information and services within an episode of care (both within and between services). By *longitudinal* co-ordination we mean the inter-linkages between staff and between agencies over a longer period of treatment, often spanning several episodes [31]. This concept has for over two decades been applied within mental health services [32,33], and is now also being applied to long-term conditions [34].

### 2.9. Efficiency

A service characteristic, which minimises the inputs needed to achieve a given level of outcomes, or which maximises the outcomes for a given level of inputs. This principle can be applied directly and with equal relevance to both physical and mental long-term conditions [35].

## 3. Innovative Forms of Patient Participation in Care

One of the major developments in mental health care in recent decades has been the direct participation of both service users and carers in planning, delivering and evaluating services [1,36,37]. Indeed in several high income countries it has become common that mental health service users’ legitimate demands for involvement in these aspects of care systems, and in exercising choice concerning specific treatments, have become progressively realised. Illustrations of these processes include decision aid tools for specific therapies [38], disclosure decisions regarding diagnoses [9], or a focus upon user-defined recovery goals [39,40]. There is also growing evidence that such forms of participation may bring measurable benefits, for example in relation to inter-personal skill training for staff [41], stigma reduction interventions [42], in developing treatment guidelines [43,44], in assessment both unmet needs [45,46] , or in employing service user researchers [47,48].

Similar developments including the participation of patients/service users are a more recent phenomenon in most areas of long-term care [49]. The National Institute of Health Report in England has categorised service user participation into four categories as: (i) none; (ii) consultation; (iii) collaboration; or (iv) service user control. As yet there are fewer published examples in the long-term care field of the more fully developed forms of service user participation. 

Nevertheless, a range of innovative service user centred modalities related to long-term conditions have emerged in recent years. For example shared care arrangements in rheumatoid arthritis encompasses treatment, ongoing education and specialist management within a shared care arrangement between patients, primary and secondary care [50,51]. Peer support programmes are more recent novel approaches, for example as applied to diabetes care [52]. In breast cancer care the development of a risk assessment and decision support aid has been shown to enhance informed and patient-centred prevention decisions [53].

There is also growing evidence that the treatment priorities and preferences of patients may differ substantially from those of health professionals. This was brought to the foreground in a series of recent publications on the outcomes of patients with chronic obstructive airway disease. Clinicians offered treatment based upon their own judgements of clinical needs, whereas patients were willing to accept such treatment recommendations provided that they were also enabled to increase their level of independence and maintain their own self-sufficiency disease [54].

A further aspect of service user or patient participation refers to patient-rated outcome measures (PROMs) and patient-generated, patient-rated outcome measures (PG-PROMs). The former have been developed in a few areas of long term conditions, such as haemophilia or scleroderma, over the last five years [55,56,57], whereas the latter have so more often been explored in relation to people with longer term mental disorders [58,59,60,61,62,63]*.*

Forms of service user participation in treatment and care have also been elaborated as collaborative care packages. The collaborative management of chronic illness model proposed by von Korff, for example, consists of four inter-linked elements: (i) collaborative definition of the problems; (ii) joint goal setting and planning; (iii) training and support in self-management; and (iv) active and sustained follow-up. This model emphasises that patients’ contribution to their self-care and their medical care are complementary [64]. An integrative overall framework of this chronic care model has undergone a transformation to the adoption of the Innovative Care of Chronic Conditions framework (ICCC) that encompasses long term conditions management with community involvement, and policy implementation to improve long-term care [65,66].

## 4. Conclusions

The lessons identified in this paper belong to two categories. First, principles which we first identified to guide mental health service organisation, all nine of which can be potentially applied to long term care as well. Second, we have outlined innovative operational aspects of service user participation, many of which were first initiated and consolidated in the mental health field, and some of which are now also being implemented in long term care. 

The implementation of both these principles and these operational innovations needs to be fine-tuned when guided by an appreciation of both the similarities and differences between long-term mental and long-term physical disorders. Examples of the similarities include: many of these conditions have relapsing and remitting clinical courses; in severe relapses individuals may need urgent care or admission; there may be fewer or less severe relapses where there is continuity of care with regular monitoring of clinical and social status; the people affected, including family members, will often benefit from psycho-social education and self-management; rates of medication adherence may be similarly low [67]; routine outcome assessment can be an important basis for ongoing care planning. 

On the other hand, perhaps the most important set of differences between long-term mental and physical disorders refer to the consequences of stigma, which can act as a powerful barrier to help seeking. Help-seeking can be conceptualised as a complex process in which health care staff practices are an important and potentially modifiable element in modeling access to healthcare [68]. The reasons why people with mental ill health sometimes avoid or delay seeking help from health services include lack of perceived need, not knowing where to go for help, perceived lack of effectiveness of treatments offered, thinking the problem will resolve itself, preferring to solve the problem on one’s own, and fear of being hospitalised against one’s will [69]. 

Recent reviews have examined the impact of stigma on access to mental health care and each concluded that it had a significant detrimental effect [70,71,72,73,74,75,76]. Evidence is lacking from low and middle income countries. In a recent systematic review most studies (69%, 99/143) were conducted in the United States or Canada; 20 were undertaken in Europe; 10 in Australia and New Zealand; 8 in Asia; and 1 in South America. A negative association was found between treatment stigma and help-seeking, effect size −0.41, −2.73 to 0.36, from 33 studies.

Perhaps paradoxically, health professionals tend to hold negative attitudes towards people with long-term mental illness [72,77,78,79,80,81,82]. Practitioners, including family physicians, report more negative ratings of people with mental illness than the general public [83,84,85,86,87,88,89,90]. The term ‘diagnostic overshadowing’ has been defined as the process by which people with mental illness receive poorer physical health care because staff mis-attribute physical symptoms to mental illness, and so under-investigate and less often treat physical disorders well [91,92]. One consequence of this, although the evidence is from high income countries, is that life expectancy is reduced by 15–20 years among people with mental disorders [93,94,95,96]. Although there are aspects of stigmatisation that apply towards some types of long-term disorder, for example against people with epilepsy or HIV/AIDS, there is evidence that this is less intensive than against people with mental illness.

This analysis leads us to the following conclusions: long term conditions, whether mental or physical, deserve a long-term commitment from the relevant health services, and indeed where continuity and co-ordination are properly funded implemented, this can ensure that the symptomatic course is more stable, quality of life is enhanced, and the clinical outcomes are more favourable. When asked, people with long-term conditions of whatever types often say that they wish to have their clinical and social needs assessed in a holistic and integrated way, rather than to have care fragmented [54,97]. Finally, we would emphasise the need to continue to strengthen the evidence-based approach to investment decisions relating to both long- term mental and physical conditions, so that cost-effective interventions can be generalised and made available to all patients able to benefit, and so that ineffective interventions and services are decommissioned. From this it follows that innovations such as self-management for long-term conditions (intended to promote autonomy) need to be subjected to the same level of rigorous scientific scrutiny as any other treatment or service intervention 03 [11,98,99,100,101,102,103].
